# Coupling Cationic g-C_3_N_4_ with Anionic Metal–Oxygen Clusters for Photocatalytic H_2_O_2_ Production

**DOI:** 10.3390/ma19204430

**Published:** 2026-10-21

**Authors:** Qiang Cui, Zilong Wang, Yu Long, Chenxi Yu, Peng Jin, Long Li, Xiangyu Meng, Yinjuan Dong

**Affiliations:** 1 State Key Laboratory of Coking Coal Resources Green Exploitation, China Pingmei Shenma Holding Group Co., Ltd., Pingdingshan 467000, Chinalilongskl@163.com (L.L.); 2 Henan Provincial Key Laboratory of Nanocomposites and Applications, Institute of Nanostructured Functional Materials, Huanghe Science and Technology College, Zhengzhou 450006, China; 3 College of Resources and Environment, Chengdu University of Information Technology, Chengdu 610225, China; wzlenvironsci@163.com (Z.W.); lyenvironsci@163.com (Y.L.); ycxenvironsci@163.com (C.Y.); 4 Pingdingshan Tian’an Coal Industry Co., Ltd., Pingdingshan 467000, China

**Keywords:** photocatalysis, hydrogen peroxide, polyoxometalates, g-C_3_N_4_, protonation

## Abstract

With growing global emphasis on green and low-carbon development, energy-related challenges have attracted heightened scholarly and public attention. Polyoxometalates (POMs)—well-defined anionic metal–oxygen clusters—exhibit exceptional capacity for rapid, reversible multi-electron transfer while maintaining structural integrity. In this study, graphitic carbon nitride (g-C_3_N_4_) was protonated using hydrochloric acid to yield the cationic derivative g-C_3_N_4_–H^+^Cl^−^. Subsequently, three nanostructured g-C_3_N_4_-based composite photocatalysts—denoted as g-C_3_N_4_-H-MMo_6_ (M = Ni, Fe, Co)—were synthesized via electrostatic assembly between the protonated support and POM anions. Comprehensive characterization was conducted to elucidate phase purity, morphology, surface properties, elemental composition, and chemical states. Under visible-light irradiation, photocatalytic H_2_O_2_ production performance was significantly enhanced: g-C_3_N_4_-H-NiMo_6_ achieved a 3.91-fold increase in activity relative to pristine g-C_3_N_4_. Catalytic efficiency followed the trend g-C_3_N_4_-H-NiMo_6_ > g-C_3_N_4_-H-FeMo_6_ > g-C_3_N_4_-H-CoMo_6_. Electrochemical tests confirmed the role of MMo_6_ (M = Ni, Fe, Co). The photogenerated electrons of g-C_3_N_4_ are transferred to the anchored POMs and reduce dissolved O_2_ to H_2_O_2_ (O_2_ + 2H^+^ + 2e^−^ → H_2_O_2_), while the photogenerated holes oxidize H_2_O to H_2_O_2_ (2H_2_O + 2h^+^ → H_2_O_2_ + 2H^+^), achieving sacrificial-agent-free H_2_O_2_ photosynthesis with an overall reaction of O_2_ + 2H_2_O → 2H_2_O_2_. Collectively, electrostatic immobilization of POMs onto protonated g-C_3_N_4_ affords robust, high-performance photocatalysts with simultaneously improved activity, stability, and structural fidelity.

## 1. Introduction

Currently, the excessive exploitation of traditional fossil fuels such as oil and natural gas is exacerbating environmental pollution and global warming, posing increasingly severe challenges to global emission reduction efforts. Reducing carbon emissions and developing a low-carbon economy have become formidable tasks for the future development of the world. In 2020, China proposed the “30·60” dual carbon goals to actively address climate issues [1].

H_2_O_2_ possesses characteristics such as high chemical reactivity, strong oxidizing-ability, and high energy density, making it widely applicable in numerous fields—including chemical synthesis, sterilization and disinfection, wastewater treatment, paper bleaching, and fuel cells [2]. Current H_2_O_2_ production faces two challenges: (i) The anthraquinone process employs noble metals such as Pd or Au-Pd as catalysts and involves multi-step reactions—including hydrogenation, oxidation, extraction, and solvent circulation—characterized by high energy consumption and susceptibility to contamination, rendering it a non-green production method. (ii) Direct synthesis of hydrogen peroxide from H_2_ and O_2_ poses an explosion hazard due to the formation of flammable H_2_/O_2_ mixtures. Therefore, developing feasible catalytic systems and efficient, low-cost, stable catalysts for H_2_O_2_ production under mild conditions is highly imperative [3]. The photocatalytic oxygen reduction reaction (ORR) for H_2_O_2_ synthesis requires only H_2_O and O_2_ as reactants and solar energy as the sole energy input in the presence of a photocatalyst. It offers advantages such as mild reaction conditions, environmentally benign operation, operational simplicity, and low energy consumption, aligning closely with sustainability objectives. As a clean chemical platform, H_2_O_2_ can serve as a key enabler for the global low-carbon energy transition and represents a viable pathway toward achieving the “dual carbon” goals [4].

The demand for green H_2_O_2_ production has driven extensive research into photocatalytic synthesis. Graphitic carbon nitride (g-C_3_N_4_) is a graphite-like layered polymer whose two-dimensional layer is built from tri-s-triazine (heptazine, C_6_N_7_) units covalently linked through nitrogen bridges (tertiary/secondary N atoms), forming an in-plane C–N network with periodic triangular nanopores; adjacent layers are stacked through weak van der Waals interactions [5]. g-C_3_N_4_ is a promising metal-free photocatalyst distinguished by its intrinsic visible-light absorption, economical synthesis, abundant raw material availability, and exceptional chemical inertness and thermal robustness under operational conditions. In 2014, Shiraishi et al. first reported the use of g-C_3_N_4_ for photocatalytic H_2_O_2_ synthesis [6]. Since then, research on g-C_3_N_4_ materials in the field of photocatalytic H_2_O_2_ production has begun to flourish. However, due to its inherent material properties, pure g-C_3_N_4_ still faces several challenges as a photocatalyst for H_2_O_2_ generation, including a relatively wide bandgap, low quantum efficiency during photocatalysis, poor separation and transfer efficiency of photogenerated electron–hole pairs, and the facile decomposition of H_2_O_2_. These drawbacks result in a persistently low efficiency of H_2_O_2_ production using pure g-C_3_N_4_, making it difficult to achieve satisfactory performance [7].

To address the aforementioned challenges, researchers have proposed various modification strategies—including elemental doping, surface functionalization, and heterojunction construction—to design highly efficient and stable g-C_3_N_4_ photocatalysts. For instance, Choi et al. in situ incorporated potassium and phosphate species into the polymeric carbon nitride framework, significantly enhancing H_2_O_2_ production while suppressing its self-decomposition [8]. Chen et al. reported a porous g-C_3_N_4_ photocatalyst doped with Na and functionalized with cyano groups; the introduction of cyano groups modulated the band structure of g-C_3_N_4_ to achieve high activity, whereas Na^+^ facilitated O_2_ reduction to generate more ·O_2_^−^, thereby improving two-electron (2e^−^) oxygen reduction reaction (ORR) selectivity [9]. Ouyang et al. designed and synthesized a novel Z-scheme heterojunction photocatalyst, which suppressed recombination of photogenerated charge carriers via interfacial electron transfer, enabling efficient photocatalytic H_2_O_2_ synthesis under ambient conditions [10]. Furthermore, employing cocatalysts—such as those catalyzing the two-electron ORR or water oxidation—provides effective active sites on the catalyst surface, enhances charge carrier migration efficiency, lowers the activation energy of catalytic reactions, improves reaction kinetics, and strengthens interfacial redox processes, thereby promoting charge separation and transfer [11]. For example, Zhang et al. grafted cationic polyimine molecules onto modified g-C_3_N_4_ via intermolecular electrostatic attraction, altering the surface charge distribution and consequently enhancing the photocatalytic activity of g-C_3_N_4_ for H_2_O_2_ generation [12]. These studies demonstrate that both structural modification of g-C_3_N_4_ and cocatalyst integration are beneficial for improving catalytic activity and selectivity. However, further research is required to elucidate the optimal cocatalyst types, their binding modes with g-C_3_N_4_, and the underlying enhancement mechanisms.

Polyoxometalates (POMs) are a class of anionic metal–oxide cluster compounds typically constructed from polyhedral metal oxide units (MO_x_) in their highest oxidation states (M = W^7+^, Mo^6+^, V^5+^, Nb^5+^, and Ta^5+^), connected via shared vertices, edges, or faces [13]. POMs offer advantages in integrating diverse molecules and are widely regarded as an excellent platform for developing functional materials. Due to their high solubility in various solvents, once dissolved, POM clusters interact electrostatically with cationic species [14], providing an intrinsic driving force that enables interactions between POMs and cations, molecules, complexes, polymers, and positively charged solid surfaces.

Anderson-type polyoxometalates (POMs) of the MMo_6_ class possess anionic structures described by the general formula [MMo_6_O_24_H_x_]^n−^, in which six edge-sharing MoO_6_ octahedra form a planar hexagonal ring surrounding a central MO_6_ octahedron; this arrangement confers D_3d_ point-group symmetry to the entire cluster [15]. As stoichiometrically defined bimetallic building blocks, MMo_6_ POMs deliver a fixed Mo:M molar ratio of 6:1—facilitating the rational design and reproducible fabrication of bimetallic nanocomposites with precisely controlled composition, crystallinity, and morphology. Furthermore, the central heterometal M can be systematically substituted with various first-row transition metals (e.g., Fe, Co, Ni, Cu, or Zn), thereby enabling deliberate modulation of electronic structure and significantly broadening the scope of electrocatalytic applications for MMo_6_-derived nanomaterials.

Based on the above, this work constructed a g-C_3_N_4_-H^+^Cl^−^ intermediate via hydrochloric acid protonation of tubular g-C_3_N_4_, and subsequently introduced three types of anionic polyoxometalates (POMs) through electrostatic interaction to fabricate three nanoscale g-C_3_N_4_-based composite photocatalysts, g-C_3_N_4_-H-MMo_6_ (M = Ni, Fe, Co). The development of this hybrid photocatalytic system provides a novel strategy for designing high-performance catalyst architectures tailored for H_2_O_2_ evolution.

## 2. Experimental Section

### 2.1. Preparation of g-C_3_N_4_

A quantity of 6 g of melamine and 8 g of urea were weighed and mixed in 75 mL of deionized water, stirred uniformly on a magnetic stirrer for 1 h, then placed into a clean liner, transferred to a 100 mL autoclave, and heated at 180 °C for 12 h in an oven. After cooling to room temperature, the product was washed with ultrapure water, centrifuged at 8000 r/min for 5 min, dried at 60 °C overnight, and then calcined in a muffle furnace at 500 °C for 4 h to obtain g-C_3_N_4_, which was ground and sealed for storage.

### 2.2. Preparation of g-C_3_N_4_-H^+^Cl^−^

The prepared 1.8 g of g-C_3_N_4_ was dispersed in 180 mL of 5 M hydrochloric acid solution. The mixture was sealed with plastic wrap and stirred at room temperature for 24 h. The resulting solid was collected by centrifugation, washed with ultrapure water until neutral pH, dried in an oven at 60 °C overnight, and ground to afford g-C_3_N_4_-H^+^Cl^−^.

### 2.3. Preparation of g-C_3_N_4_-H-MMo_6_ (M = Ni, Fe, Co)

The Anderson-type polyoxometalates (POMs) used, denoted as MMo_6_ (M = Fe, Co, Ni), were synthesized according to the published literature [15] and are represented by the ammonium salts (NH_4_)_3_[FeMo_6_O_24_H_6_]·7H_2_O, (NH_4_)_4_[CoMo_6_O_24_H_6_]·xH_2_O, and (NH_4_)_4_[NiMo_6_O_24_H_6_]·5H_2_O. Quantities of 0.5 g of g-C_3_N_4_-H^+^Cl^−^ and 0.05 g of the corresponding MMo_6_ POM (M = Ni, Fe, or Co) were dispersed in 50 mL of ultrapure water, heated in an oil bath at 100 °C for 10 h, and then cooled to room temperature. The resulting solid was collected by centrifugation, washed thoroughly with ultrapure water, dried in an oven at 60 °C overnight, ground into a fine powder, and stored in a sealed container.

### 2.4. Photocatalytic H_2_O_2_ Production Activity Test

Photocatalytic H_2_O_2_ production activity tests were conducted as follows: 15 mg of catalyst was placed in 15 mL of ultrapure water. An LED light source (Beijing Perfectlight, Beijing, China, PLS-LED100C, 100 mW cm^−2^, λ = 420 nm) was used, and the suspension was exposed to ambient air under stirring. The irradiation time was varied, while the light power was kept constant at 100 W. At hourly intervals, 1 mL aliquots were withdrawn and immediately sealed for subsequent analysis. Each sample was centrifuged, and 500 µL of the supernatant was mixed with 4 mL of detection solution. The mixture was allowed to stand for 10 min to complete the colorimetric reaction. The absorbance of the liberated iodine was measured at 352 nm using a Shimadzu UV-2700 UV–Vis spectrophotometer (Kyoto, Japan), and the H_2_O_2_ concentration was quantified by reference to a calibration curve, thereby reflecting the photocatalytic activity of the catalyst.

The quantification of H_2_O_2_ is based on the well-established iodometric colorimetric method. In the presence of ammonium molybdate as the catalyst, H_2_O_2_ oxidizes the iodide ions (I^−^) provided by KI to iodine (I_2_): H_2_O_2_ + 2I^−^ + 2H^+^ → I_2_ + 2H_2_O; the generated I_2_ immediately combines with the excess I^−^ to form the triiodide complex (I_3_^−^): I_2_ + I^−^ ⇌ I_3_^−^ (overall: H_2_O_2_ + 3I^−^ + 2H^+^ → I_3_^−^ + 2H_2_O) [16]. The triiodide ion exhibits a characteristic absorption maximum at 352 nm, and its absorbance follows the Beer–Lambert law.

## 3. Results and Discussion

### 3.1. Catalyst Structure Characterization

**Scheme 1:**
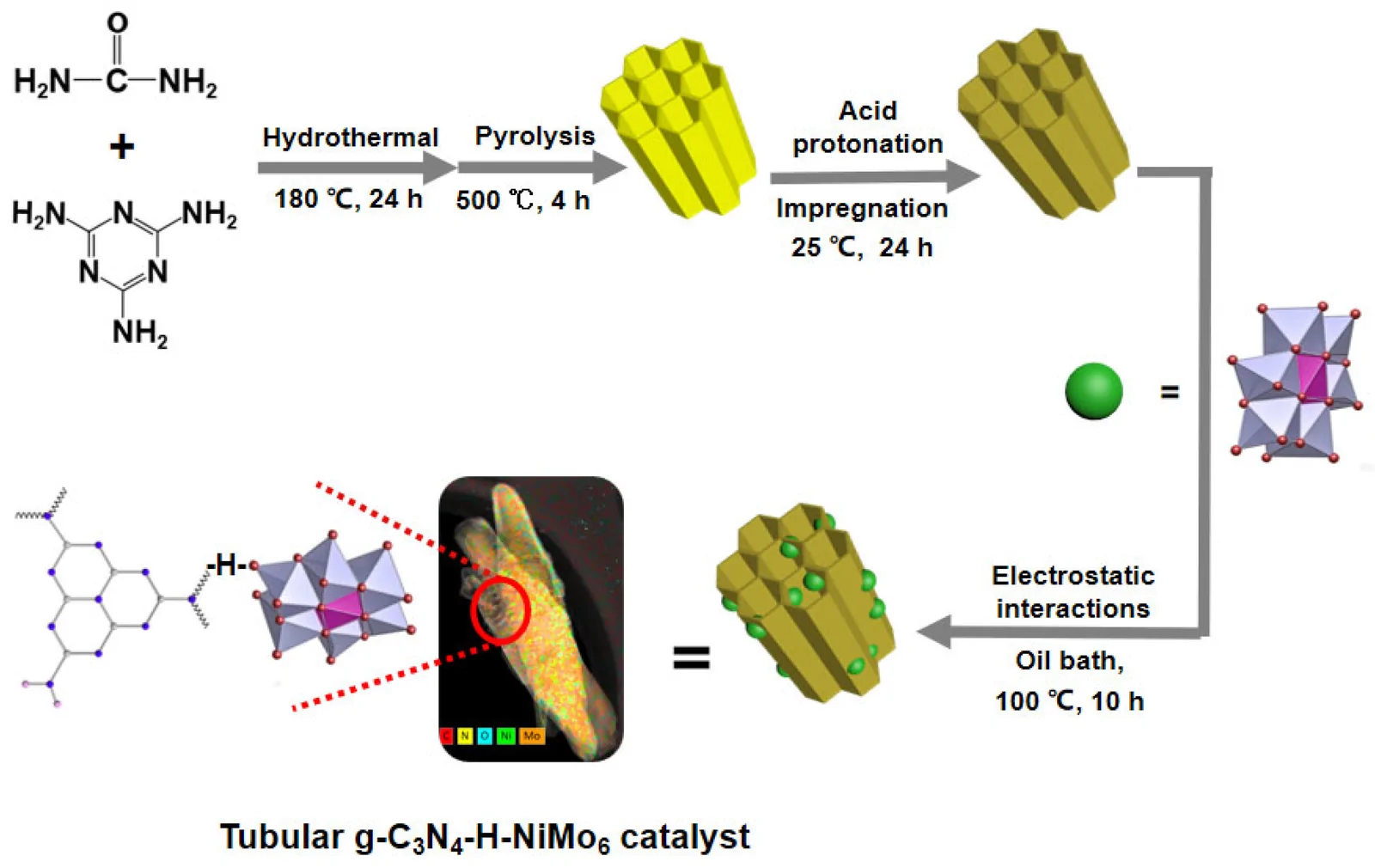
Schematic illustration for the formation process over g-C_3_N_4_-H-MMo_6_ catalysts.

As schematically outlined in Scheme 1, the synthesis of MMo_6_ POM/g-C_3_N_4_-H^+^Cl^−^ composites proceeds via a multi-step protocol. First, hydrothermal treatment of melamine and urea at 180 °C affords a hydrogen-bonded supramolecular (melamine–cyanuric acid) rod-like precursor. During the subsequent calcination at 500 °C, urea decomposes to release gases (NH_3_, CO_2_) that act as a porogen, while the rod-like precursor is converted into porous tubular g-C_3_N_4_ [17,18]. The resulting g-C_3_N_4_ is then protonated by dispersing it in 5 M aqueous HCl; under these strongly acidic conditions, protonation occurs preferentially at terminal amino groups and interlayer sites, while Cl^−^ anions simultaneously intercalate into the interlamellar spaces. After centrifugation, thorough washing with deionized water until neutral pH (~7), and vacuum drying, the protonated material—designated g-C_3_N_4_-H^+^Cl^−^—is obtained. Finally, pre-synthesized, water-soluble MMo_6_ POMs (M = Ni, Fe, Co) are introduced into an aqueous dispersion of g-C_3_N_4_-H^+^Cl^−^, and the mixture is subjected to mild hydrothermal crystallization at 100 °C. This step enables electrostatic self-assembly between the positively charged g-C_3_N_4_-H^+^Cl^−^ matrix and the anionic MMo_6_ POM species, resulting in a homogeneous heterogeneous composite material.

**Figure 1:**
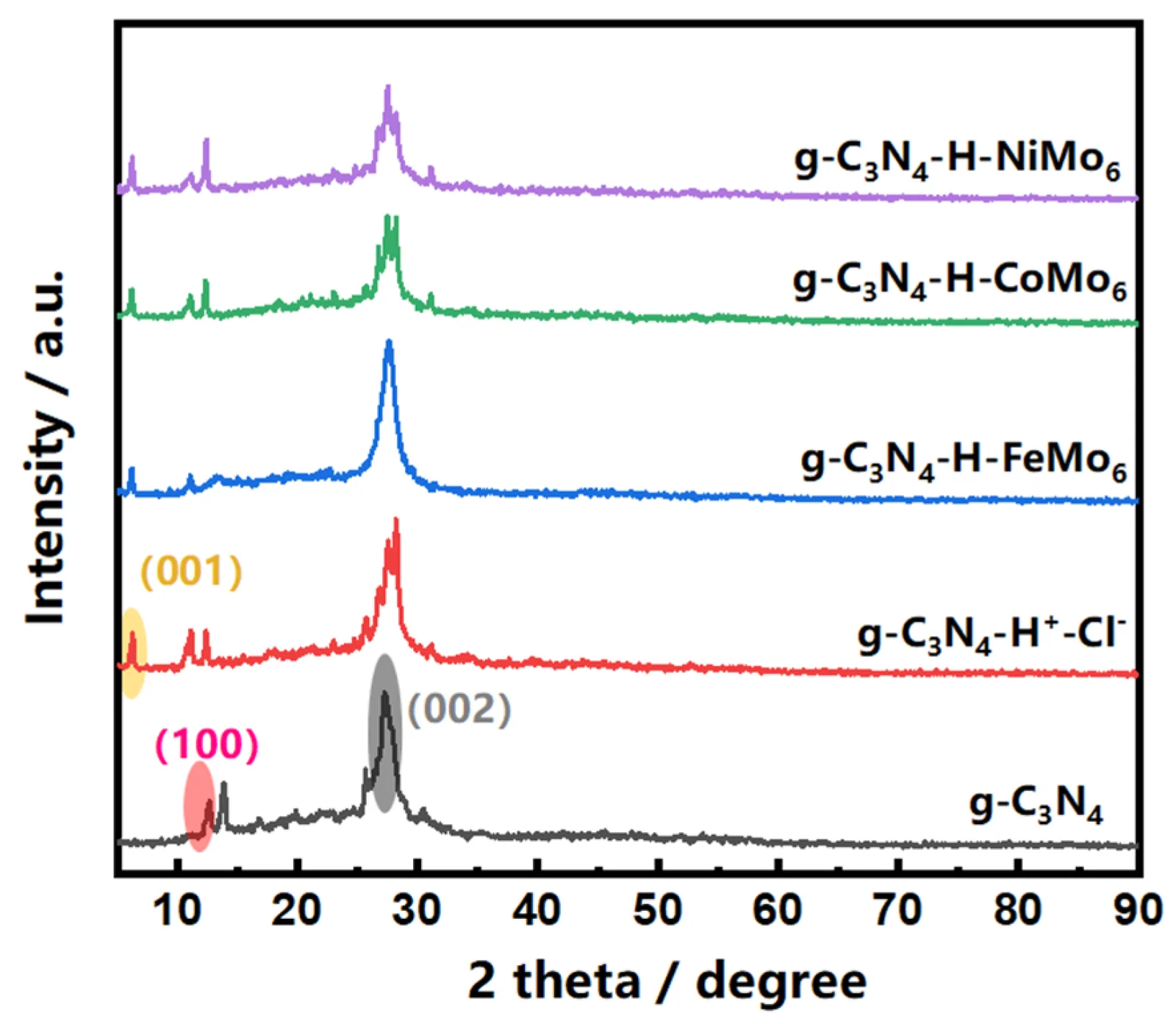
XRD patterns of g-C_3_N_4_, g-C_3_N_4_-H^+^-Cl^−^, g-C_3_N_4_-H-FeMo_6_, g-C_3_N_4_-H-CoMo_6_, and g-C_3_N_4_-H-NiMo_6_.

As shown in Figure 1, the phase and crystal structures were characterized by X-ray diffraction (XRD). All materials exhibit a typical diffraction peak at ~13°, assigned to in-plane structural ordering associated with the triazine ring motif. The diffraction peak at ~27° is attributed to interlayer stacking of aromatic systems [19]. In the XRD patterns of g-C_3_N_4_-X (X = HCl, FeMo_6_, CoMo_6_, NiMo_6_), in addition to the characteristic (100) reflection at ~13.1° (d ≈ 0.68 nm) and the (002) interlayer-stacking reflection at ~27.5° (d ≈ 0.32 nm) of g-C_3_N_4_ (JCPDS No. 87-1526), a new reflection at 2θ ≈ 6° (d ≈ 1.47 nm) appearing after HCl protonation is attributed to the expansion of the interlayer periodicity induced by protonation/intercalation during the acid treatment. This assignment is consistent with the observation of a new low-angle diffraction peak at 6.2° (d = 1.421 nm) in g-C_3_N_4_ nanosheets, indexed to the (001) lattice plane [20]. The slightly larger d-spacing in our sample (1.47 nm vs. 1.42 nm) is ascribed to a higher degree of proton intercalation/hydration under our treatment conditions. Furthermore, several weak fine reflections superimposed on the broad (002) peak were observed for the POM-modified samples; they are assigned to the characteristic reflections of the Anderson-type POM crystallites (e.g., FeMo_6_ at 2θ ≈ 27.7°, CoMo_6_ at 2θ ≈ 26.8° and NiMo_6_ at 2θ ≈ 28.6°), whose low intensity is due to the small POM loading and the high dispersion of the nanoscale POM crystallites. Meanwhile, the rest of the non-peak regions showed an XRD pattern similar to that of g-C_3_N_4_. This demonstrates the successful preparation of composite catalysts combining graphitic phase g-C_3_N_4_ and POMs. Moreover, since the crystal structure of the composite material showed no significant change, it suggests that the composite material still retains the structural characteristics of g-C_3_N_4_ [21].

**Figure 2:**
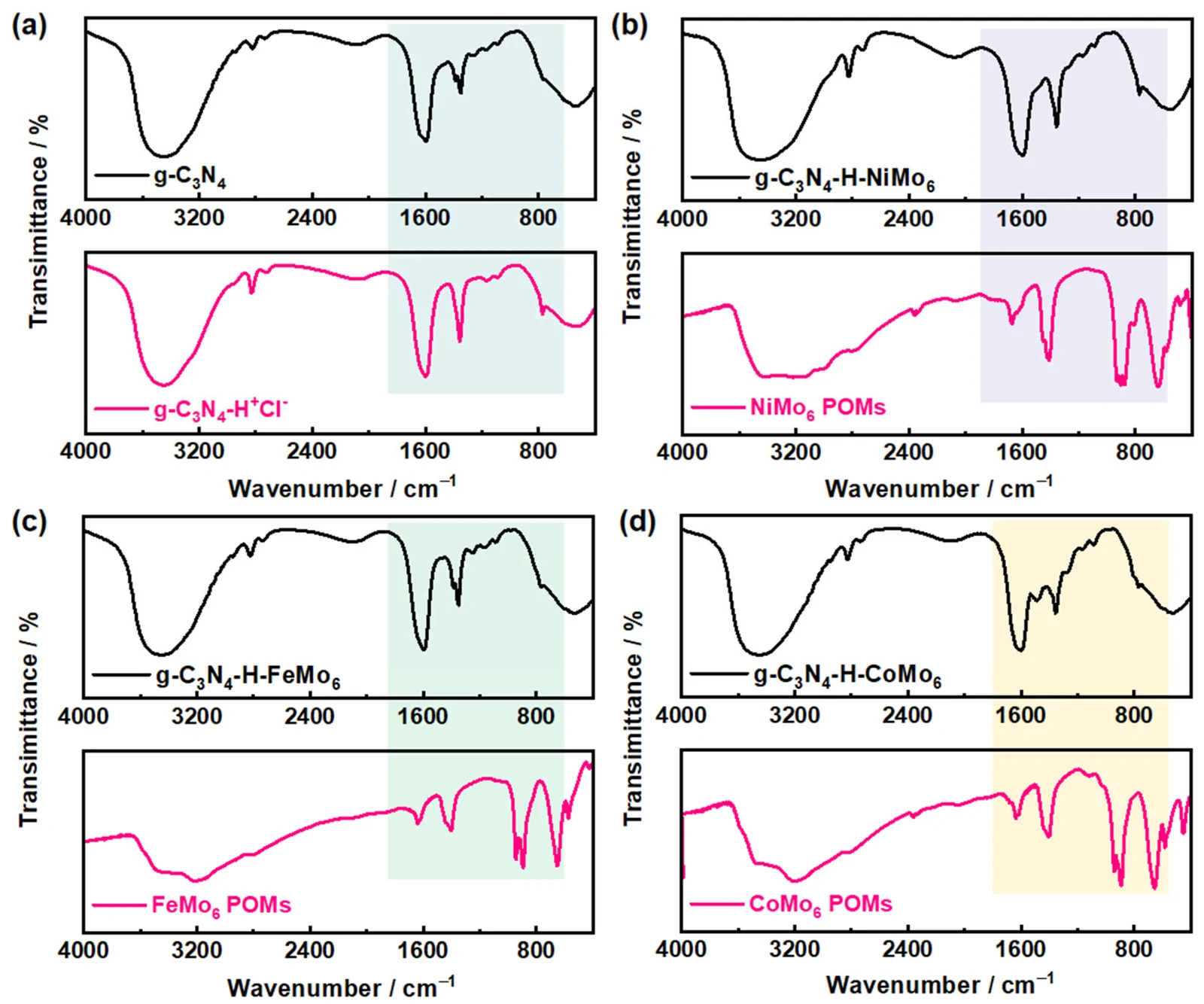
FT-IR spectra of (**a**) g-C_3_N_4_ and g-C_3_N_4_-H^+^Cl^−^; (**b**) g-C_3_N_4_-H-FeMo_6_ and FeMo_6_ POM; (**c**) g-C_3_N_4_-H-CoMo_6_ and CoMo_6_ POM; (**d**) g-C_3_N_4_-H-NiMo_6_ and NiMo_6_ POM.

As shown in Figure 2, the Fourier-transform infrared spectroscopy (FT-IR) spectra of g-C_3_N_4_, g-C_3_N_4_-H^+^Cl^−^, g-C_3_N_4_-H-FeMo_6_, g-C_3_N_4_-H-CoMo_6_, g-C_3_N_4_-H-NiMo_6_, and FeMo_6_, CoMo_6_, and NiMo_6_ POMs are presented. In Figure 2a, compared to g-C_3_N_4_, the protonated sample g-C_3_N_4_-H^+^Cl^−^ does not exhibit any distinct peaks. In Figure 2b–d, all composite samples, except for the FeMo_6_, CoMo_6_, and NiMo_6_ POMs, display the characteristic absorption peaks of g-C_3_N_4_. The peak at 803 cm^−1^ and the peaks in the range of 1150–1700 cm^−1^ correspond to the stretching vibrations of the triazine ring and the C≡N heterocycle, respectively [21]. The broad peak in the range of 3000–3500 cm^−1^ is attributed to surface-adsorbed water and the stretching vibration of N-H bonds [22]. The FT-IR spectra show the characteristic bands of the Anderson-type anion: terminal Mo=O stretching at 1000–800 cm^−1^, bridging Mo–O–Mo stretching at 750–550 cm^−1^, Mo–O (H)–M stretching below 450 cm^−1^, together with O–H/N–H stretching at 3600–2800 cm^−1^ [15]. The characteristic peaks of FeMo_6_, CoMo_6_, and NiMo_6_ POM materials overlap to some extent with those of the catalyst materials, making it difficult to observe significant changes in peak shape when the loading amount is small.

The formation of pore channels helps increase the specific surface area, indicating that the catalyst’s activity is likely improved compared to the raw g-C_3_N_4_ material. Additionally, the image also shows the typical layered structures of the graphitic-phase g-C_3_N_4_, indicating that some portion of the g-C_3_N_4_-H-NiMo_6_ was not completely transformed into the columnar structure.

The g-C_3_N_4_-H-NiMo_6_ material was selected for scanning electron microscopy (SEM) testing, and Figure S1 shows the results. It can be observed that part of the g-C_3_N_4_-H-NiMo_6_ material has formed a columnar structure, with white spots attached, which are speculated to be the attachment of POMs. The outer surface of the columnar structure exhibits some tubular collapse, suggesting potential pore channels within the columnar structure, where the walls might be too thin, leading to damage and the formation of voids during preparation [23]. The formation of pore channels helps increase the specific surface area, indicating that the catalyst’s activity is likely improved compared to that of the raw g-C_3_N_4_ material.

Additionally, the image also shows typical layered structures of graphitic phase g-C_3_N_4_, indicating that some portion of the g-C_3_N_4_-H-NiMo_6_ was not completely transformed into the columnar structure. The coexistence of the two morphologies originates from the different self-assembly behaviour of the two precursors during the hydrothermal step [24]. The melamine and the cyanuric acid generated from urea form hydrogen-bonded supramolecular aggregates that grow into rod-like precursors, which are converted into the rod/tube-like g-C_3_N_4_ upon calcination. The urea-derived fraction that does not participate in this rod-like supramolecular assembly directly condenses into bulk sheet-like g-C_3_N_4_ domains.

**Figure 3:**
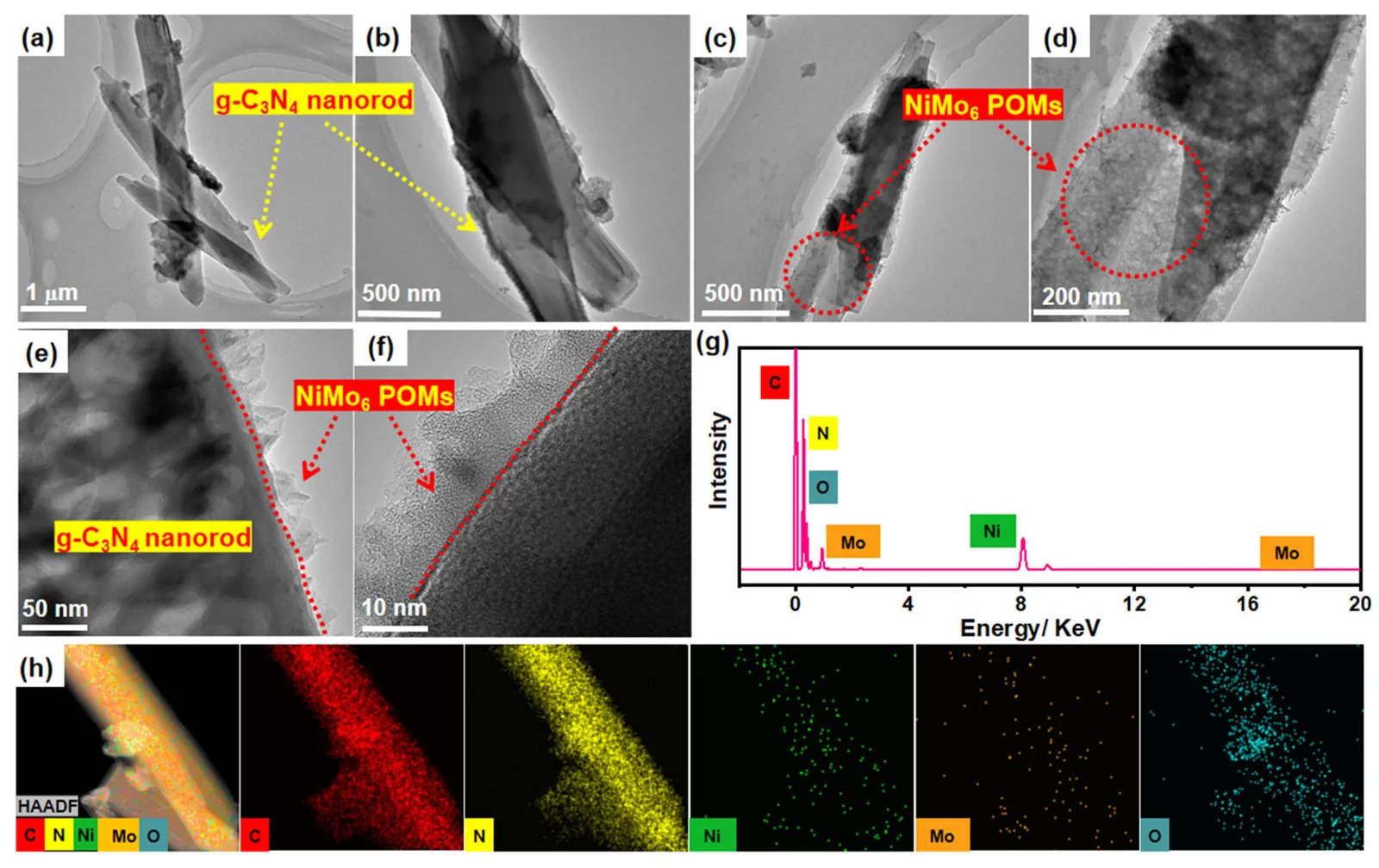
Morphological characteristics of g-C_3_N_4_-H-NiMo_6_ catalysts: (**a**–**d**) TEM images and (**e**,**f**) the corresponding high-resolution TEM images; (**g**) Energy dispersive X-ray analysis; (**h**) Element mapping.

As shown in Figure 3a–d, g-C_3_N_4_-H-NiMo_6_ exhibits a unique nanorod morphology. Figure 3a,b indicate that g-C_3_N_4_ forms a nanorod structure. In Figure 3c,d, at higher magnification, numerous NiMo_6_ POM nanoparticles are clearly visible, uniformly dispersed on the surface of g-C_3_N_4_ nanorods, confirming successful NiMo_6_ POM incorporation and the formation of the target composite material. High-resolution transmission electron microscopy (HRTEM) images in Figure 3e,f further verify the precise construction of the g-C_3_N_4_-H-NiMo_6_ homogeneous–heterogeneous composite electrocatalyst. Energy-dispersive X-ray spectroscopy (EDX, Figure 3g) and elemental distribution mapping (Figure 3h) collectively demonstrate that C, N, Ni, Mo, and O are uniformly distributed throughout the catalyst structure, while the overall content of NiMo_6_ POM is relatively low, which explains the weak signals observed in the XRD and FT–IR patterns.

**Figure 4:**
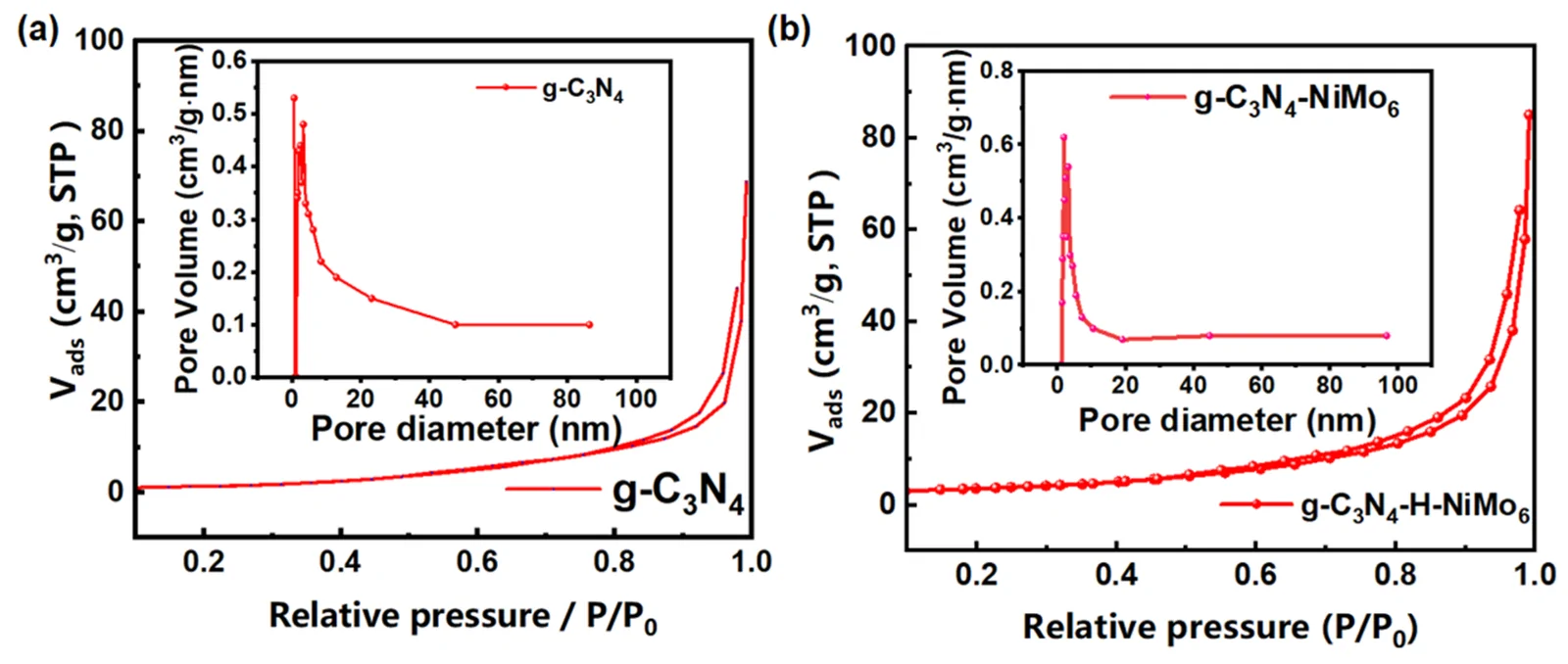
N_2_ adsorption–desorption isotherms of the prepared (**a**) g-C_3_N_4_ and (**b**) g-C_3_N_4_-H-NiMo_6_ catalysts. The inset shows the corresponding pore-size distribution curves.

To investigate the adsorption performance of the catalyst materials, this experiment utilized Brunauer–Emmett–Teller (BET) analysis to determine the specific surface area and pore structure of the materials. The adsorption isotherm under nitrogen was employed to characterize the pore size distribution and specific surface area of the photocatalysts. The initial graphitic phase g-C_3_N_4_ material and the NiMo_6_ POM composite catalyst material were selected as representatives. As shown in Figure 4, the isotherms for both materials are typical Type IV with H_3_ hysteresis loops, indicating interactions between the adsorbent and adsorbate, accompanied by capillary condensation and confirming the presence of mesopores. In the pore size distributions of both materials, distinct peaks are observed, with pore sizes concentrated in the ranges of 13–70 Å and 13–61 Å, respectively. After measurement and calculation, the specific surface area, total pore volume, and average pore diameter were obtained. The specific surface area of g-C_3_N_4_-H-NiMo_6_ was 13.052 m^2^/g, larger than that of g-C_3_N_4_ (5.623 m^2^/g). The single-point total pore volume of g-C_3_N_4_-H-NiMo_6_ was 0.1322 cm^3^/g, larger than that of g-C_3_N_4_ (V = 0.1069 cm^3^/g), indicating enhanced adsorption capacity under identical conditions. Based on the test results, the graphitic-phase g-C_3_N_4_ material combined with POMs exhibits an increased specific surface area, which is beneficial for exposing active sites and enhancing catalytic performance [25,26].

**Figure 5:**
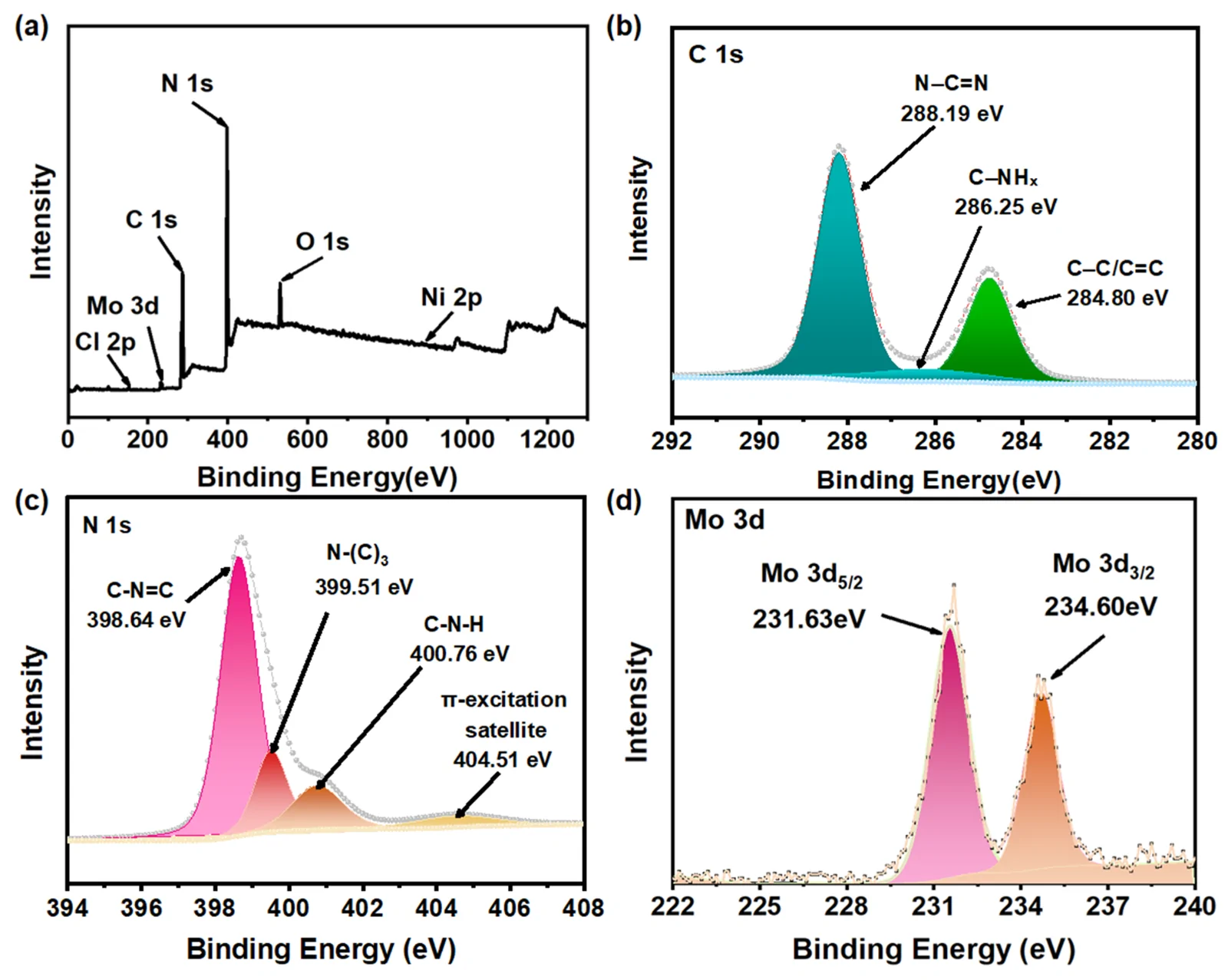
(**a**) Survey XPS spectrum of g-C_3_N_4_-H-NiMo_6_; (**b**) C 1s XPS spectrum; (**c**) N 1s XPS spectrum; and (**d**) Mo 3d XPS spectrum.

X-ray photoelectron spectroscopy (XPS) characterization was used to investigate the chemical states and elemental composition of g-C_3_N_4_-H-NiMo_6_. Figure 5 shows the XPS survey spectrum of g-C_3_N_4_-H-NiMo_6_, as well as the C 1s, N 1s, Ni 2p, and Mo 3d core-level spectra. The survey spectrum confirms the presence of the main elements C, N, Ni, and Mo in the material. It also reveals O and Cl; the former is attributed to adsorbed water and carbon dioxide on the surface of the material, while the latter originates from incomplete conversion of the protonated precursor to the POM composite material.

The C 1s XPS was deconvoluted into three distinct peaks, assigned to C–C/C=C (284.80 eV), C–NH_x_ (286.25 eV), and N–C=N (288.19 eV), the latter corresponding to the carbon atoms in the tri-s-triazine framework and exhibiting the highest intensity [27,28]. The N 1s X-ray photoelectron spectrum (XPS) was deconvoluted into four well-resolved peaks, assigned to C–N=C (398.64 eV), N–(C)_3_ (399.51 eV), C–N–H (400.76 eV), and a π-excitation satellite peak at 404.51 eV [29,30]. Various nitrogen configurations may play distinct roles in catalytic processes. Pyridinic nitrogen introduces defects and vacancies into the electronic structure of carbon-rich materials, increasing the charge carrier density and thereby enhancing electronic conductivity, which facilitates rapid electron transfer. Pyrrolic nitrogen improves hydrophilicity and electrical conductivity, and serves as an anchoring site for constructing more efficient composite catalysts.

In the Mo 3d spectrum (Figure 5d), two fitted peaks at binding energies of 231.63 eV and 234.60 eV correspond to Mo 3d_5/2_ and Mo 3d_3/2_, respectively, confirming Mo incorporation into the same precursor. The XPS analysis confirms that both the pristine g-C_3_N_4_ and the g-C_3_N_4_-H-NiMo_6_ catalysts contain their intended elements and exhibit characteristic chemical bonding, demonstrating the feasibility of integrating the NiMo_6_ POM with g-C_3_N_4_. Since hydrogen atoms bond with other elements in the material and lack core-level electrons suitable for XPS excitation, they cannot be detected by XPS [31]. The measured content of elemental analysis (ICP-OES) is Ni: 0.24 wt%/Mo: 2.4 wt%. The measured molar ratios of Ni and Mo contents agree well with the theoretical value of 1:6.

### 3.2. Photocatalytic Activity Performance

**Figure 6:**
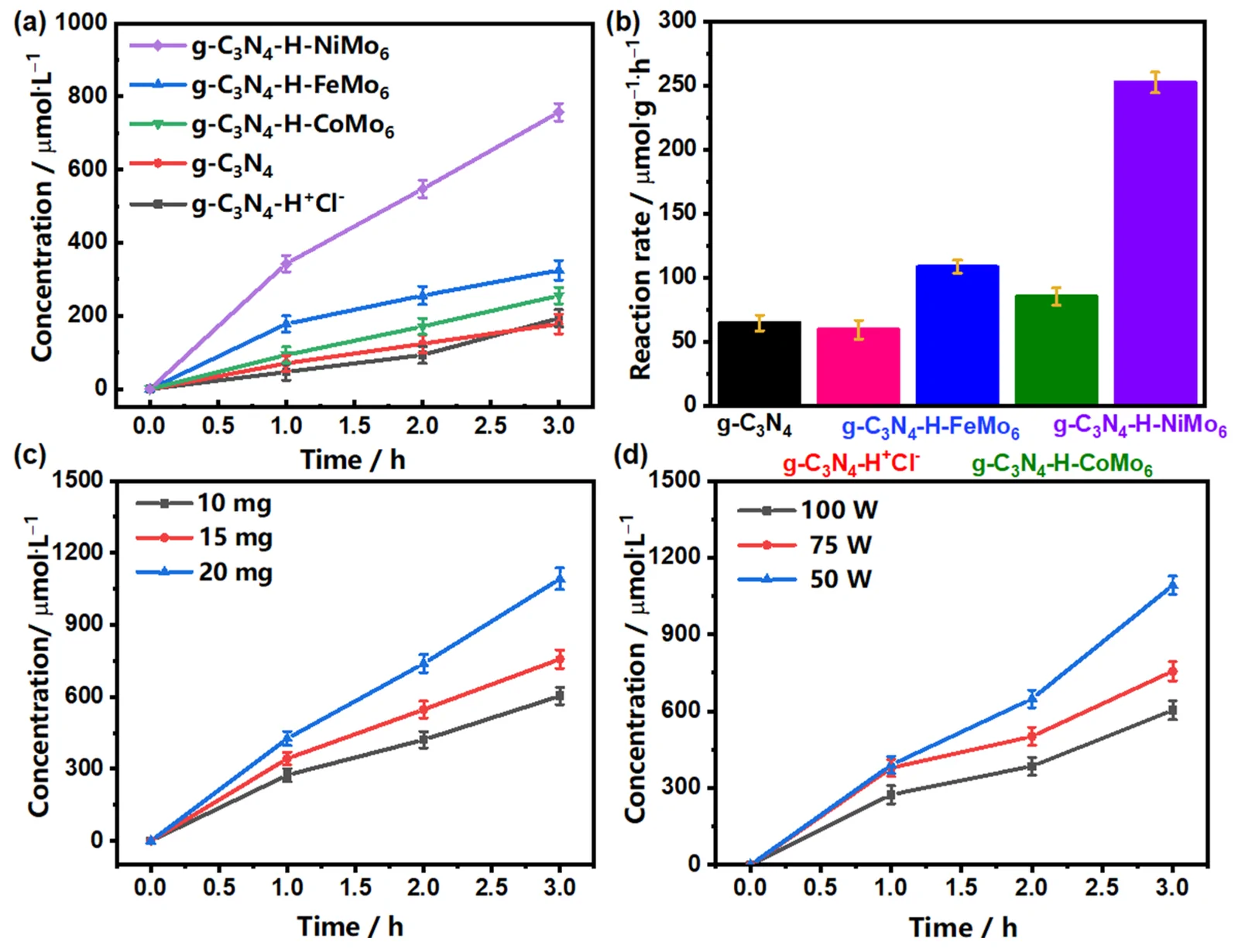
(**a**) Photocatalytic H_2_O_2_ production; and (**b**) Bar graph of reaction rates for g-C_3_N_4_, g-C_3_N_4_-H^+^Cl^−^and g-C_3_N_4_-H-FeMo_6_; g-C_3_N_4_-H-CoMo_6_; g-C_3_N_4_-H-NiMo_6_ catalysts; (**c**) Kinetic curve of H_2_O_2_ production changes in g-C_3_N_4_-H-NiMo_6_ within 3 h under different catalyst dosages; (**d**) Kinetic curve of H_2_O_2_ production changes in g-C_3_N_4_-H-NiMo_6_ within 3 h under different intensities of LED lamp.

Activity tests were conducted at room temperature under irradiation from a 100 W LED lamp with a sampling interval of 1 h. The photocatalytic H_2_O_2_ synthesis performance of g-C_3_N_4_; g-C_3_N_4_-H^+^Cl^−^; g-C_3_N_4_-H-FeMo_6_; g-C_3_N_4_-H-CoMo_6_; and g-C_3_N_4_-H-NiMo_6_ was evaluated over 3 h. The absorbance data obtained from the experiments were converted to H_2_O_2_ concentrations using a calibration curve (Table S1 and Figure S2). As shown in Figure 6a,b, under visible light irradiation, the photocatalytic H_2_O_2_ synthesis rates for g-C_3_N_4_ and g-C_3_N_4_-H^+^Cl^−^ were 64.5 μmol·g^−1^·h^−1^ and 59.4 μmol·g^−1^·h^−1^, respectively. The rates for g-C_3_N_4_-H-FeMo_6_, g-C_3_N_4_-H-CoMo_6_, and g-C_3_N_4_-H-NiMo_6_ were 108.3 μmol·g^−1^·h^−1^, 85.2 μmol·g^−1^·h^−1^, and 252.3 μmol·g^−1^·h^−1^, respectively. Compared to pristine g-C_3_N_4_, the H_2_O_2_ production rates of the Fe-, Co-, and Ni-doped POM composite catalysts were 1.7-fold, 1.3-fold, and 3.9-fold higher, respectively. The photocatalytic H_2_O_2_ synthesis rate of hydrochloric acid-treated g-C_3_N_4_ showed a slight increase, while those of g-C_3_N_4_ doped with FeMo_6_ POMs, CoMo_6_ POMs, or NiMo_6_ POMs were further enhanced. The data in the figure indicate that combining POMs with g-C_3_N_4_ effectively improves catalyst activity [32]. Among the g-C_3_N_4_ materials modified with different POMs, g-C_3_N_4_-H-NiMo_6_ exhibited the highest photocatalytic H_2_O_2_ synthesis rate and the highest activity.

The experiment investigating the effect of catalyst loading on the photocatalytic H_2_O_2_ production over g-C_3_N_4_-H-NiMo_6_ was conducted at room temperature, using a 100 W LED lamp and 15 mL of ultrapure water. Catalyst loadings were varied at 10 mg, 15 mg, and 20 mg to evaluate their influence on the reaction rate. The average photocatalytic H_2_O_2_ synthesis rates over 3 h were 201.3 μmol·g^−1^·h^−1^, 252.3 μmol·g^−1^·h^−1^, and 363.9 μmol·g^−1^·h^−1^ for catalyst loadings of 10 mg, 15 mg, and 20 mg, respectively. Compared with the 10 mg loading, the 3 h average rates increased by approximately 25% and 80% for 15 mg and 20 mg, respectively. Relative to the 15 mg loading, the rate at 20 mg increased by 44%. It can be observed that the increment in catalytic rate per additional 5 mg of catalyst is non-uniform. Moreover, as shown in Figure 6c, the H_2_O_2_ concentration profile is non-linear. This deviation from linearity is attributed to unavoidable experimental variables, including: (1) real-time fluctuations in dissolved O_2_ concentration; (2) minor variations in LED irradiance due to power supply instability; and (3) slight ambient temperature drifts. Nevertheless, it is evident that catalyst loading significantly influences the photocatalytic reaction rate.

As shown in Figure 6d, the experiment investigating the effect of LED irradiance on the photocatalytic H_2_O_2_ production over g-C_3_N_4_-H-NiMo_6_ was conducted at room temperature using 15 mg of catalyst and 15 mL of ultrapure water. LED lamp powers were varied at 100 W, 75 W, and 50 W to evaluate their influence on the reaction rate. The results show that at irradiance levels corresponding to 100 W, 75 W, and 50 W LED input power, the average photocatalytic H_2_O_2_ synthesis rates over 3 h were 252.3 μmol·g^−1^·h^−1^, 205.8 μmol·g^−1^·h^−1^, and 179.7 μmol·g^−1^·h^−1^, respectively. Compared with the 100 W condition, the 3 h average rates decreased by approximately 19% and 29% at 75 W and 50 W, respectively. Relative to the 75 W condition, the rate at 50 W decreased by 13%. It can be observed that the reduction in catalytic rate per 25 W decrease in input power is non-uniform. Additionally, as shown in Figure 6d, the H_2_O_2_ concentration profile is non-linear, with contributing factors identical to those identified in the catalyst loading study. Nevertheless, it is evident that LED lamp input power significantly influences the photocatalytic reaction rate.

### 3.3. Mechanism Investigation

**Figure 7:**
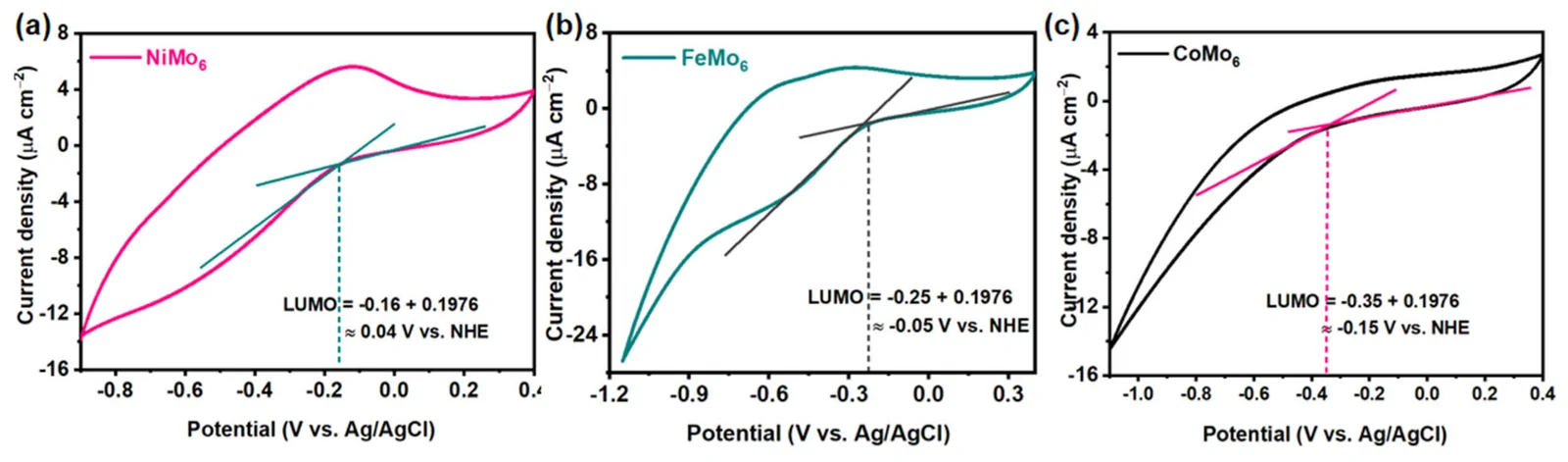
Cyclic voltammetry of (**a**) NiMo_6_, (**b**) FeMo_6_; and (**c**) CoMo_6_. Test conditions: 5 mg POMs dissolved in 50 mL water, scan rate of 50 mV/s, working electrode as glassy carbon electrode, reference electrode as Ag/AgCl, counter electrode as Pt foil.

To further understand the significant differences in H_2_O_2_ evolution activity among NiMo_6_; FeMo_6_; and CoMo_6_ (Figure 7a–c), we measured the energy levels of these molecular POMs using electrochemical cyclic voltammetry. As shown in Figure 7a, both NiMo_6_; FeMo_6_; and CoMo_6_ exhibit reduction peaks, with calculated LUMO (Lowest Unoccupied Molecular Orbital) levels of 0.04 V (vs. NHE (Normal Hydrogen Electrode)); −0.05 V (vs. NHE); and −0.15 V (vs. NHE), respectively. At the same concentration, NiMo_6_ shows the highest peak intensity, indicating that NiMo_6_ has the best electron-capturing ability.

**Figure 8:**
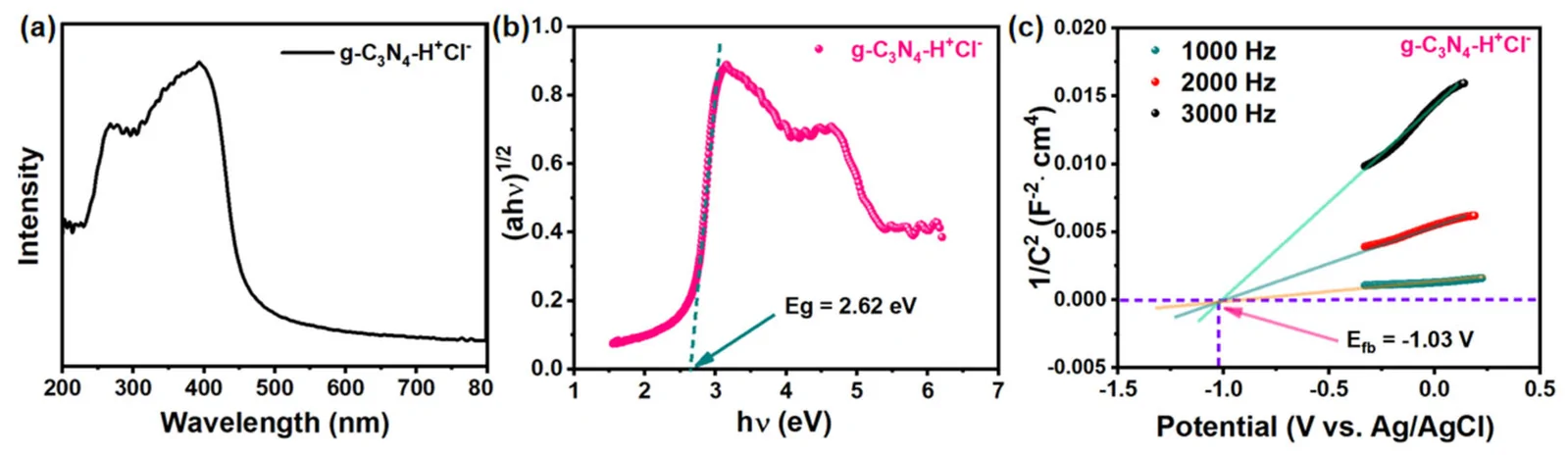
(**a**) UV-vis absorption spectrum; (**b**) Tauc plots of (αhν)^2^ versus hν; and (**c**) Mott–Schottky plots of the g-C_3_N_4_-H^+^Cl^−^.

UV–vis diffuse reflectance spectroscopy (DRS) reveals a high absorption response of g-C_3_N_4_ (Figure 8a). According to the Kubelka–Munk equation [33], the band gap of g-C_3_N_4_-H^+^Cl^−^ was calculated to be 2.62 eV (vs. NHE) (Figure 8b). The conductivity type of g-C_3_N_4_-H^+^Cl^−^ was evaluated using a three-electrode system at frequencies of 1000 Hz, 2000 Hz, and 3000 Hz via Mott–Schottky plots [34] (Figure 8c). The positive slope of the C^−2^–E curve indicates that g-C_3_N_4_-H^+^Cl^−^ is an n-type semiconductor. The flat-band potential (E_fb_) of g-C_3_N_4_-H^+^Cl^−^ was determined to be −1.03 V (vs. Ag/AgCl) by Mott–Schottky analysis. Therefore, the conduction band (CB) potential of g-C_3_N_4_ is −1.03 eV, corresponding to −0.83 V vs. the standard hydrogen electrode (NHE) (E_NHE_ = E_Ag/AgCl_ + E^θ^_Ag/AgCl_; E^θ^_Ag/AgCl_ = 0.1976 V) [35].

**Scheme 2:**
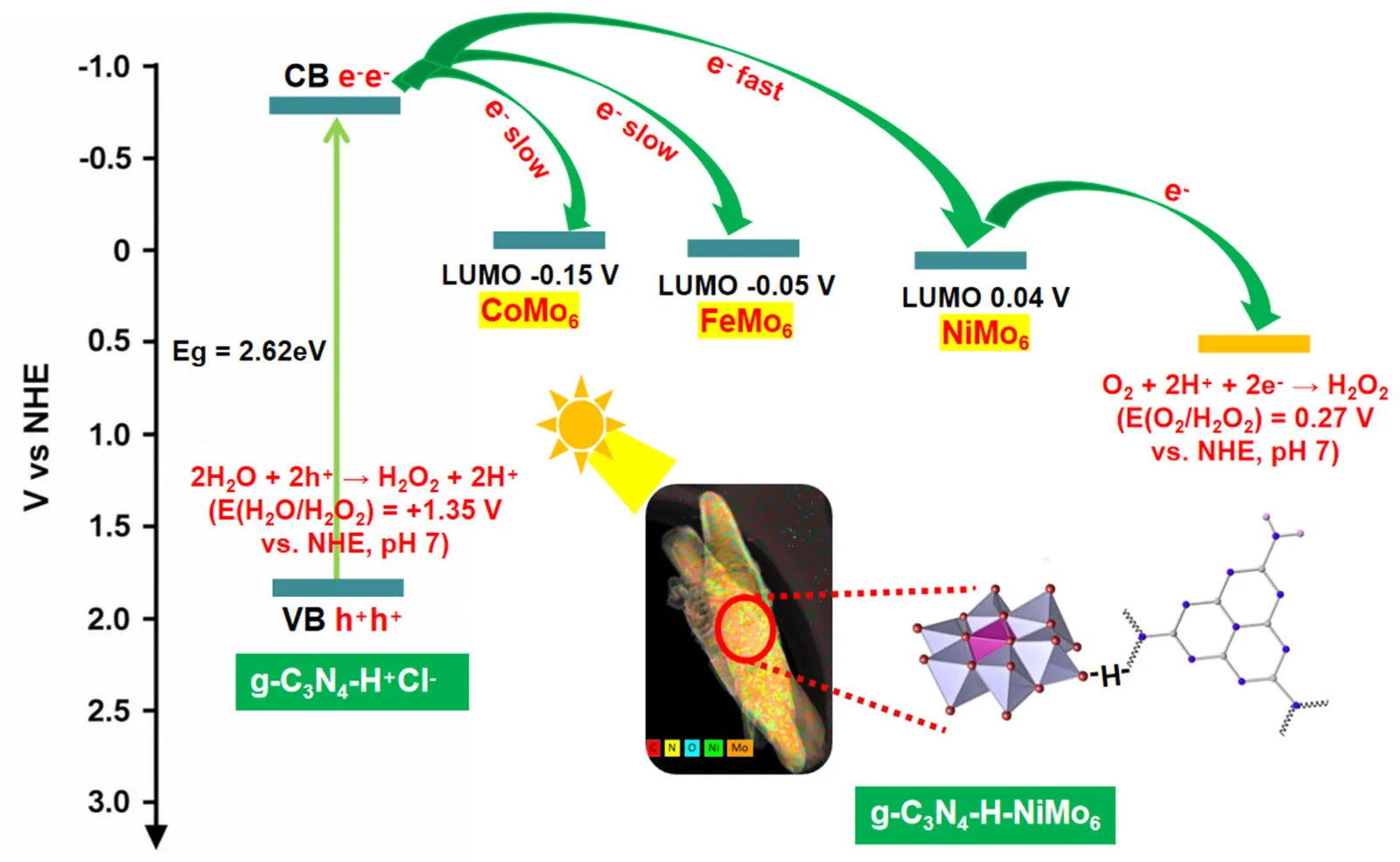
Schematic diagram of electron transition energy levels over g-C_3_N_4_-H-NiMo_6_ catalyst.

Based on the above data, we have drawn the electron transfer mechanism diagram shown in Scheme 2. It can be observed that NiMo_6_ possesses the most suitable LUMO level [36], which facilitates the transfer of photogenerated electrons from the conduction band of g-C_3_N_4_ to protons in solution, thereby exhibiting high H_2_O_2_ evolution activity. Upon light irradiation, g-C_3_N_4_ generates electron–hole pairs. The electrons in the CB are transferred to the Anderson-type POMs (NiMo_6_, LUMO ≈ +0.04 V vs. NHE), which then act as electron mediators to reduce O_2_ to H_2_O_2_: O_2_ + 2H^+^ + 2e^−^ → H_2_O_2_ (E(O_2_/H_2_O_2_) = +0.27 V vs. NHE, pH 7). Meanwhile, the holes accumulated in the VB of g-C_3_N_4_ (VB edge ≈ +1.79 V vs. NHE) oxidize H_2_O to H_2_O_2_: 2H_2_O + 2h^+^ → H_2_O_2_ + 2H^+^ (E(H_2_O/H_2_O_2_) = +1.35 V vs. NHE, pH 7). This oxidation is thermodynamically feasible because the VB edge of g-C_3_N_4_ is more positive than the H_2_O/H_2_O_2_ redox potential. Therefore, the reduction of O_2_ and the accompanying oxidation of H_2_O constitute two coupled half-reactions, giving the overall reaction O_2_ + 2H_2_O → 2H_2_O_2_ without any sacrificial electron donor.

## 4. Conclusions

As green and low-carbon development gains increasing attention, energy issues have gradually attracted more and more public concern. Polyoxometalates (POMs), as anionic metal–oxygen clusters, can undergo rapid and reversible multi-electron transfer reactions without altering their structure. In this work, g-C_3_N_4_ was protonated with hydrochloric acid to construct g-C_3_N_4_-H^+^Cl^−^ material. Subsequently, POMs were immobilized onto the protonated substrate via electrostatic interactions to prepare three nanosized g-C_3_N_4_-based composite catalysts [g-C_3_N_4_-H-MMo_6_ (M = Ni, Fe, Co)]. Their phase structures and morphologies were characterized, and their photocatalytic activities—as well as the key influencing factors—were systematically investigated. SEM and TEM analyses revealed that the g-C_3_N_4_-H-NiMo_6_ composite exhibits a nanorod morphology. Their phase structures and morphologies were characterized, and their catalytic activities along with influencing factors were investigated. SEM and TEM analysis revealed that the g-C_3_N_4_-H-NiMo_6_ composite catalyst exhibits a columnar structure. FTIR results confirmed that the prepared composites retain the characteristic structural features of g-C_3_N_4_. BET measurements demonstrated that g-C_3_N_4_-H-NiMo_6_ possesses a larger specific surface area than pristine g-C_3_N_4_, suggesting enhanced adsorption capacity. XPS was employed to probe the elemental composition and chemical states of the materials. Photocatalytic H_2_O_2_ production experiments showed that the activity of g-C_3_N_4_-H-NiMo_6_ (252.3 μmol·g^−1^·h^−1^) was 3.91-fold higher than that of pure g-C_3_N_4_ (64.5 μmol·g^−1^·h^−1^). The catalytic performance of the composite catalysts varied with the metal identity in the POMs, following the order: g-C_3_N_4_-H-NiMo_6_ > g-C_3_N_4_-H-FeMo_6_ > g-C_3_N_4_-H-CoMo_6_. NiMo_6_, with its optimal LUMO energy level, superior reduction capability, and strong adsorption affinity, functions as an efficient cocatalyst for photocatalytic H_2_O_2_ evolution. In summary, the composite catalysts fabricated via electrostatic coupling of protonated g-C_3_N_4_ with POMs exhibit both enhanced photocatalytic performance and improved operational stability.

## Data Availability

The original contributions presented in this study are included in the article/Supplementary Materials. Further inquiries can be directed to the corresponding authors.

## Supplementary Materials

The following supporting information can be downloaded at: https://www.mdpi.com/article/10.3390/ma19204430/s1, Figure S1: SEM images of g-C_3_N_4_-H-NiMo_6_ at magnifications corresponding to (a) 5 μm, (b) 2 μm, (c) 1 μm, and (d) 500 nm; Figure S2: Calibration plot of H_2_O_2_ Production; Table S1: Preparation of the H_2_O_2_ standard solutions (final volume 10 mL; stock concentration ≈ 20 mmol L^−1^).
