# Supplementary material for: Coupling Cationic g-C3N4 with Anionic Metal–Oxygen Clusters for Photocatalytic H2O2 Production
Source: Materials (Basel). 2026 Oct 21;19(20):4430. doi: 10.3390/ma19204430 (PMC13707213; doi:10.3390/ma19204430)
Supplement: Supplementary file 1 [file materials-19-04430-s001.zip › materials-4609933-supplementary.pdf]

Supplementary Materials

Coupling Cationic g-C_3N_4 with Anionic Metal-Oxygen Clusters for Photocatalytic H_2O_2 Production

Qiang Cui[1,#], Zilong Wang[3,#], Yu Long[3,#], Yu Chenxi, Peng Jin[1,4*], Long Li[1], Xiangyu Meng[2,*], Yinjuan Dong[3,*]

[1] State Key Laboratory of Coking Coal Resources Green Exploitation, China Pingmei Shenma Holding Group Co., Ltd., Pingdingshan, Henan 467000, PR China

[2] Henan Provincial Key Laboratory of Nanocomposites and Applications, Institute of Nanostructured Functional Materials, Huanghe Science and Technology College, Zhengzhou, Henan 450006, PR China

[3] College of Resources and Environment, Chengdu University of Information Technology, Chengdu, 610225, China

[4] Pingdingshan Tian'an Coal Industry Co., Ltd., Pingdingshan, Henan 467000

[#]These authors contributed equally

* Correspondence: jinpengxx@163.com (P. Jin); 202206132@hhstu.edu.cn (X. Meng); dyjchem@163.com (Y. Dong)

1 Experimental Section

1.1 Experimental Reagents

Melamine was purchased from Tianjin Damao Chemical Reagent Factory; urea from Chengdu Kelong Chemical Co., Ltd.; potassium iodide from Aladdin Company; ammonium molybdate tetrahydrate from Tianjin Hengxin Chemical Co., Ltd.; 30% hydrogen peroxide from Xiamen Anyongbo Technology Co., Ltd.; 37% hydrochloric acid from Guangzhou Howei Technology Co., Ltd.; potassium bromide from Tianjin Botianshengda Technology Development Co., Ltd.

1.2 Characterization

Ultrapure deionized water (18.2 MΩ·cm) was produced via a Millipore Milli-Q water purification system. The crystalline structure of the synthesized materials was characterized by X-ray diffraction (XRD, DX-2700BH) employing Cu K_α radiation (40 kV, 40 mA), with data collected over a 2θ range of 5–85°. X-ray photoelectron spectroscopy (XPS, Shimadzu Axis Supra) equipped with monochromatic Al K_α radiation (1486.6 eV) was utilized to analyze chemical states and surface composition, where binding energies were referenced to the adventitious carbon C 1s peak at 284.8 eV for calibration. Morphological analysis was performed using scanning electron microscopy (SEM, Quattro S) in conjunction with high-resolution transmission electron microscopy (HRTEM, FEI Talos F200S). Functional groups were identified through Fourier-transform infrared spectroscopy (FT-IR, Nicolet iS50). Textural properties, including specific surface area and pore size distribution, were derived from N_2 physisorption isotherms measured at 77 K using an SSA-4000 analyzer (Beijing Piodde Electronic Technology Co., Ltd.), with the Brunauer–Emmett–Teller (BET) method applied for surface area calculation and the Barrett–Joyner–Halenda (BJH) method used for pore size distribution analysis.

1.3 Standard Curve Preparation

The absorbance of the liberated iodine was measured at 352 nm using a Shimadzu UV-2700 UV–Vis spectrophotometer, and the H_2O_2 concentration was quantified by reference to a calibration curve, thereby reflecting the photocatalytic activity of the catalyst. First, The H_2O_2 concentration was determined by an iodometric colorimetric method. A H_2O_2 stock solution was first prepared by adding 100 μL of 30% H_2O_2 (≈ 9.8 mol L^{-1}) to 50 mL of deionized water, giving a stock concentration of ≈ 20 mmol L^{-1} (100×10^{-6} L × 9.8 mol L^{-1} ÷ 50×10^{-3} L ≈ 19.6 mmol L^{-1}). A series of standard solutions with concentrations of 0, 0.10, 0.20, 0.30, 0.40 and 0.50 mmol L^{-1} was then prepared from this stock solution by the dilution equation $C_1V_1 = C_2V_2$ (final volume 10 mL each), as listed in Table S1.

The detection reagent is prepared by dissolving 809.7 mg of potassium ioide and 15.1 mg of ammonium molybdate tetrahydrate in 50 mL of pure water, followed by continuous

stirring for 1 hour. For measurement, 500 μL of each H_2O_2 standard solution is mixed with 4 mL of the detection reagent and thoroughly combined. The mixture is then incubated in the dark for 10 minutes (wrapped in tin foil), after which the absorbance at 352 nm is recorded. Finally, the test results are plotted the standard curve (See **Fig,S2**).

2. Results and Discussion

Table S1. Preparation of the H_2O_2 standard solutions (final volume 10 mL; stock concentration ≈ 20 mmol L^{-1}).

C_{stock} (mmol L^{-1})	Volume of 20 mmol L^{-1} stock	Water to 10 mL
0	0	10.00 mL
0.10	50 µL	9.95 mL
0.20	100 µL	9.90 mL
0.30	150 µL	9.85 mL
0.40	200 µL	9.80 mL
0.50	250 µL	9.75 mL

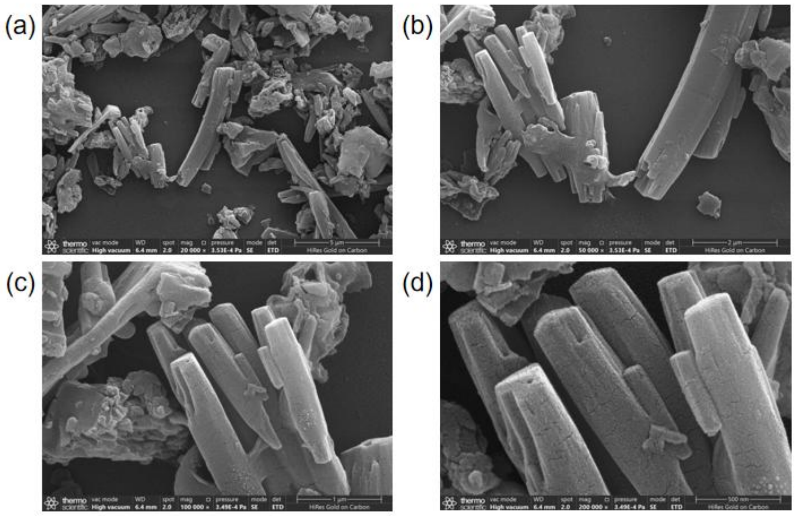

Figure S1 SEM images of g-C_3N_4-H-$NiMo_6$ at magnifications corresponding to (a) 5 μm, (b) 2 μm, (c) 1 μm, and (d) 500 nm.

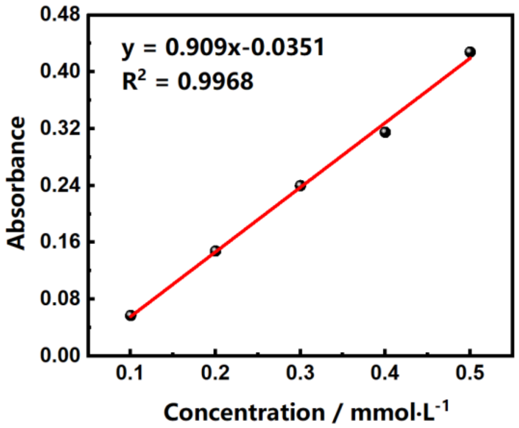

Figure S2 Calibration plot of H_2O_2 Production.
